# Primary open angle glaucoma in a Caucasian population is associated with the *p53* codon 72 polymorphism

**Published:** 2009-09-22

**Authors:** Christopher L. Daugherty, Hilda Curtis, Tony Realini, Judie F. Charlton, Sepideh Zareparsi

**Affiliations:** 1 Department of Ophthalmology, West Virginia University, Morgantown, WV; 2 Department of Biochemistry, West Virginia University, Morgantown, WV

## Abstract

**Purpose:**

Apoptosis has been implicated as the mechanism for retinal ganglion cell death in primary open-angle glaucoma (POAG), a complex neurodegenerative disease. There have been inconsistent reports regarding increased risk of POAG and a polymorphism (Arg72Pro) within the tumor suppressor gene, *p53*. The goal of this study was to examine the role of this polymorphism in susceptibility to POAG in a Caucasian population from the United States.

**Methods:**

We generated genotypes in 191 unrelated Caucasian POAG patients and 167 unrelated Caucasian controls for the following polymorphisms within *p53*: rs1042522 (Arg72Pro), rs17878362 (16 bp Ins/Del), and rs1800371 (Pro47Ser) by PCR amplification followed by restriction digestion and sequence analysis.

**Results:**

There was a significant difference in genotypic frequencies for rs1042522 (Arg72Pro) between POAG patients and controls (χ^2^= 9.56, p=0.008). Individuals who were homozygous for the arginine allele have a 1.9 fold significantly increased risk of developing glaucoma (95%CI: 1.16-2.82, p=0.01). Interestingly, we found that the frequency of the arginine allele was even higher in the normal-tension glaucoma (NTG) subtype compared to high-tension POAG (0.81 versus 0.76).

**Conclusions:**

Our preliminary results indicate that the arginine variant of rs1042522 within *p53* is associated with increased risk of POAG. This variant has increased apoptotic potential, thus the retinal ganglion cells in carriers of the arginine allele may have greater susceptibility to apoptosis.

## Introduction

Glaucoma, the second leading cause of blindness in the world, includes a group of eye disorders characterized by visual field defects, retinal ganglion cell death, and progressive degeneration of the optic nerve. Adult-onset primary open-angle glaucoma (POAG) represents the most prevalent form of glaucoma, and affects 33 million individuals worldwide [1]. It is recognized that POAG is a multi-factorial disorder involving the role of multiple genes as well as environmental factors. The major risk factors for the development of POAG include advanced age, race, elevated intraocular pressure (IOP), and family history. Genome-wide linkage analysis in families with an autosomal dominant form of POAG have resulted in identification of eight chromosomal regions that harbor genes involved in POAG [2-9]. To date, mutations in three genes (myocilin [*MYOC*], optineurin [*OPTN*], and WD repeat domain 36 [*WDR36*]) have been found to be associated with POAG [8,10,11]. However, these mutations only account for 3-5% of all POAG cases [12]. Thus, the genetic basis of the majority of POAG cases remains unknown.

The death of retinal ganglion cells in glaucoma has been the subject of extensive research, which has implicated apoptosis as the pathway for programmed cell death (reviewed in [13]). One of the important regulatory proteins in apoptosis is the tumor suppressor protein p53, known as the “guardian of the genome”. p53 can promote apoptosis through a transcription-dependent mechanism or independent of transcriptional regulation. Under normal conditions, p53 levels and activity are tightly regulated. Upon diverse forms of cellular stress the steady state levels and transcriptional activity of *p53* are considerably increased. Moreover, it has been shown that *p53* is active in retinal ganglion cells. Specifically, elevated levels of p53 have been detected within the inner retina after transient retinal ischemia [14]. In heterozygote mice with a *p53* null mutation, there was significant resistance to ischemia and preservation of the inner retinal layer [14].

Interestingly, *p53* has been implicated in development of POAG. There have been inconsistent reports regarding increased risk of glaucoma and genetic variations within *p53*. An association was originally detected between POAG and a SNP in exon 4 of *p53* at codon 72 (rs1042522-Arg72Pro) in a Chinese population [15]. However, two additional groups did not observe this association in a group of Indian and Tasmanian POAG patients [16,17]. Examination of a 16 base pair (bp) insertion/deletion polymorphism located within *p53* intron 3 (rs17878362) has provided suggestive evidence that a specific haplotype based on this variation and the *p53* exon 4 SNP is associated with POAG [16,18]. However, it should be noted that each of these studies examined different ethnicities from outside of United States. Data from the Hapmap project for this SNP confirms differences in genotypic and allelic frequencies between different ethnic groups.

The goal of this study was to examine the association between POAG and several SNPs within *p53* in a large Caucasian population recruited at the West Virginia University Eye Institute that have the unique feature of representing the Appalachian population. In our cohort, there is evidence for an association between POAG risk and the *p53* Arg72Pro polymorphism based on the results provided below.

## Methods

This study was reviewed and approved by the West Virginia University Institutional Review Board. All subjects provided written informed consent and authorization to use protected health information. For this retrospective case-control association study, subjects with primary open-angle glaucoma and subjects without glaucoma were recruited from the glaucoma and the comprehensive clinics of the WVU Eye Institute. All subjects underwent ophthalmic examination including assessment of visual acuity and intraocular pressure (IOP) by Goldmann tonometry, slit-lamp inspection of the anterior chamber, and dilated fundoscopic evaluation of the optic nerve and retina. The medical records of all subjects were carefully reviewed to determine historical IOP measurements and any historical diagnosis of glaucoma or suspicion of glaucoma. All subjects also completed a questionnaire detailing their ophthalmic and medical history as well as the ophthalmic history of their family including the presence of glaucoma in their siblings, parents, children, grandparents, aunts, and uncles. Subjects with glaucoma or suspected glaucoma underwent additional evaluation including assessment of central corneal thickness, gonioscopy, and standard automated perimetry using the SITA-Standard 24-2 or 30-2 testing algorithm on the Humphrey Field Analyzer II. To minimize perimetry learning-effect artifacts, all subjects were experienced with automated perimetry, and only reliable visual fields (defined as fewer than 20% fixation losses, false-positives, and false-negatives with no obvious artifacts such as those attributable to trial lens rim or lid droop) were included for analysis.

Subjects were classified as having primary open-angle glaucoma based on the presence of all of the following: anterior chamber angles open by gonioscopy, reproducible visual field defects (arcuate, nasal step, or temporal wedge defects or generalized constriction) on standard perimetry, and glaucomatous optic neuropathy (focal thinning or notching of the Neuroretinal rim, generalized enlargement of the optic cup, presence of a disc hemorrhage, focal nerve fiber layer bundle defects, or inter-eye asymmetry of the cup-disc ratio exceeding 0.2). Glaucoma subjects with IOP never being recorded over 21 mmHg were considered normal tension glaucoma subjects as a subgroup of the open angle glaucoma patients. Subjects were classified as non-glaucomatous if all of the following criteria were met: no history of IOP above 21 mmHg, no findings consistent with conditions associated with secondary glaucoma (such as pigment dispersion syndrome, pseudoexfoliation syndrome, uveitis, or anterior segment neovascularization); and no evidence of glaucomatous optic neuropathy as described above. Subjects with some but not all of the primary open-angle glaucoma criteria were classified as glaucoma suspects, as were subjects with IOP above 21 mmHg in the absence of either visual field defects or glaucomatous optic neuropathy; these glaucoma suspects were excluded from analysis.

All subjects underwent venipuncture to obtain a blood sample for genetic analysis. Genomic DNA was extracted from peripheral blood leukocytes by the Gentra Puregene Blood kit (Qiagen, Valencia, CA) according to the manufacturer’s protocol. We obtained genotypes for the following polymorphisms within *p53*: 1) rs1042522 (Arg72Pro, G→C, exon 4); 2) rs17878362 (16 bp Ins/Del, intron 3); and 3) rs1800371 (Pro47Ser, C→T, exon 4). In each case, the polymorphism was detected by PCR followed by restriction digestion analysis (Table 1). In some samples, genotypes were confirmed by direct sequencing.

**Table 1:** Primer sequence and restriction enzymes used for genotyping the polymorphisms within *p53*.

**SNP**	**Distance**	**Enzyme**	**F-primer**	**R-primer**
rs1042522	0	BstUI	GTGGGAAGCGAAAATTCCAT	GCCAGGCATTGAAGTCTCAT
rs1800371	-76	MspI	GACCTGTGGGAAGCGAAAAT	GAGCAGCCTCTGGCATTCT
rs17878362	-172	BstUI	GTGGGAAGCGAAAATTCCAT	GCCAGGCATTGAAGTCTCAT

For each polymorphism, genotypic distributions were examined for significant departure from the Hardy-Weinberg equilibrium by χ^2^ analysis for patients and controls. Genotypic and allelic frequencies were compared between POAG cases and controls by χ^2^ analysis. Odds ratios were calculated using a conditional logistic model, adjusting for age and sex. All analyses were conducted using SPSS software (Release 15.0; SPSS Inc., Chicago, IL).

## Results

Our cohort consisted of 167 unrelated Caucasian controls and 191 unrelated Caucasian POAG patients, of whom 52 had NTG. Subject characteristics are summarized in Table 2. The majority of subjects were of European descent and had the unique feature of representing the Appalachian population. Although patients were older than controls in our study, the controls were older than the average age of diagnosis and were still free of any signs of POAG. Cases were screened for the presence of the 3 most frequent mutations in *MYOC*: Gly364Val, Gln368Stop, and Tyr437His and no carriers were detected for these mutations.

**Table 2:** Subject characteristics.

**Demographic features**	**Controls**	**All POAG**	**POAG-HTG**	**POAG-NTG**
N	167	191	139	52
Females	63%	48%	42%	65%
Age at Inclusion	60.3±12.0	67.5±12.5	66.6±12.6	69.8±12.0
Age at Diagnosis		58.8±18.9	58.1±20.7	60.9±12.5
Family history of POAG	18%	35%	36%	31%

We examined genotypic and allelic frequencies for the Arg72Pro SNP (rs1042522) within *p53* in our cohort, which were in Hardy-Weinberg equilibrium in both POAG patients and controls. There is a significant difference in genotypic frequencies for rs1042522 between POAG patients and controls (χ^2^= 9.56, p=0.008; Table 3). There were 49% homozygotes for arginine, 43% hetreozygotes and 8% homozygotes for proline among controls as compared to 65% homozygotes for arginine, 29% hetreozygotes and 6% homozygotes for proline among POAG patients. The allelic frequencies were 71% for arginine and 29% for proline in the controls compared to 80% for arginine and 20% for proline in POAG. The frequency of the arginine allele (G) is significantly increased in POAG patients compared to controls (p≤0.01). Interestingly, the frequency of the arginine allele is even higher in the NTG subtype compared to the high-tension POAG (0.84 versus 0.78).

**Table 3:** Genotypic and allele frequencies in POAG patients and controls for rs1042522.

**Genotype/Allele**	**Controls**	**All POAG**	**POAG-HTG**	**POAG-NTG**
G/G	0.49	0.65	0.64	0.69
G/C	0.43	0.29	0.29	0.29
C/C	0.08	0.06	0.07	0.02

G (Arg)	0.71	0.80	0.78	0.84
C (Pro)	0.29	0.20	0.22	0.16

**Figure 1:**
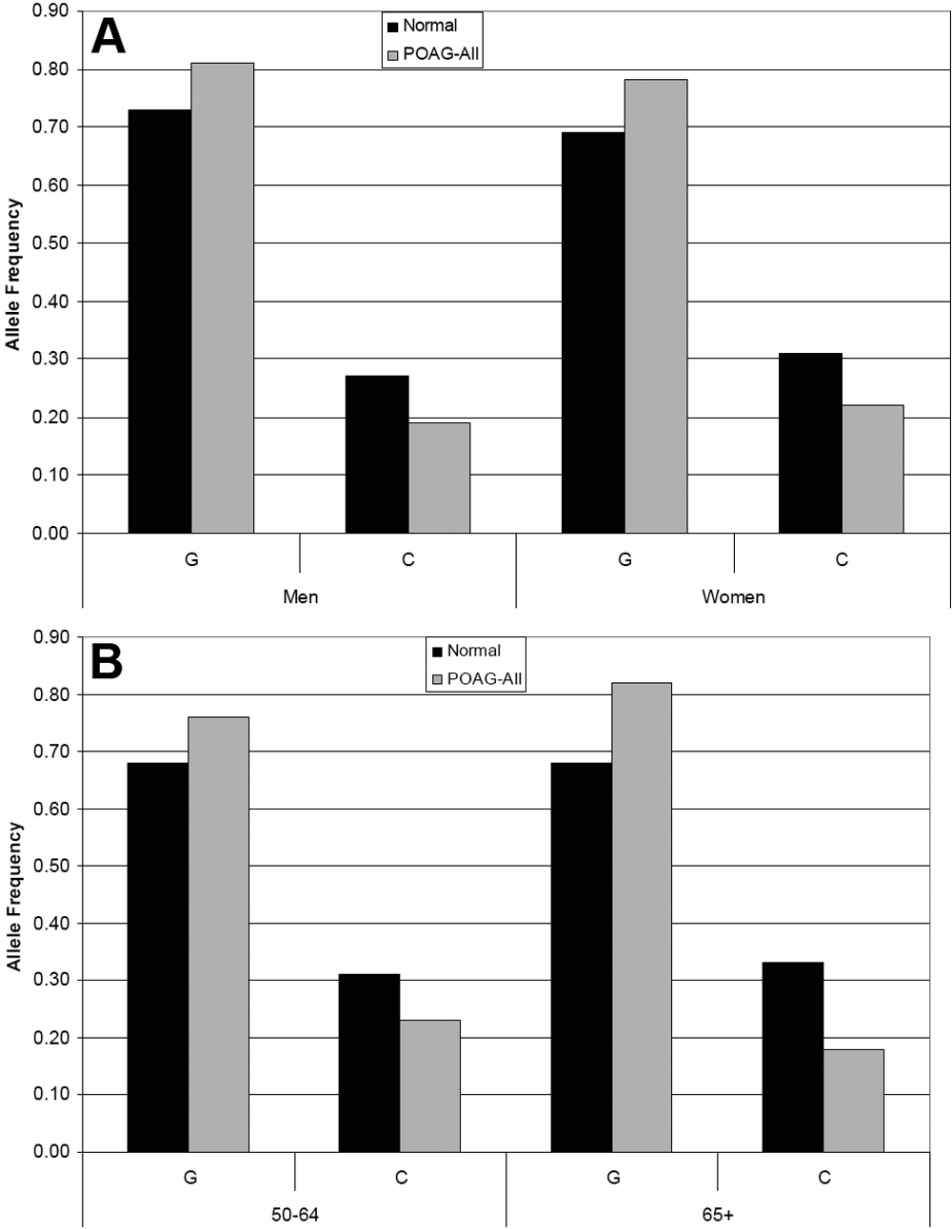
Allele Frequencies for rs1042522 in POAG patients and controls by sex and age. **A**: The frequency of the arginine allele (G) and proline allele (C) in men and women. In both men and women, the frequency of the arginine allele (G) is higher in POAG patients than controls. **B**: Subjects were divided into two age groups: group 1 from 50-64 years old and group 2 from 65-99 years old. In both age groups, the frequency of the arginine allele (G) is higher in POAG patients than controls.

Individuals who were homozygous for the arginine allele had a significant 1.95 fold increased risk of developing POAG than those who are heterozygous or lack the arginine allele (95%CI: 1.27 – 2.98, p=0.002). Since there are differences in age and proportion of females between patients and controls, we examined allele frequencies by sex and by age groups. The frequency of the arginine allele (G) was increased in POAG patients compared to controls in both men and women (Figure 1A). For age comparisons, we grouped the subjects into 2 age groups: group 1 from 50-64 years old and group 2 from 65-99 years old. In both age groups, the frequency of the arginine allele (G) was increased in POAG patients compared to controls (Figure 1B). The age- and sex-adjusted odds ratio indicate that homozygotes for the arginine allele still had a significant 1.92 fold increased risk of developing POAG (95%CI: 1.22–3.0, p=0.005). We wondered if the association would be stronger in POAG patients with a positive family history of POAG. The allelic frequencies were 85% for arginine and 15% for proline in those with a positive family history compared to 77% for arginine and 23% for proline in those without a family history. Although the frequency of the arginine allele was slightly elevated, it was not significant.

Next, we examined rs17878362 which is a 16 bp Ins/del that is 172 bp away from rs1042522. There were no significant differences in genotypic or allelic frequencies for this SNP (Table 4). However, there was a specific haplotype (rs1042522: arginine and rs17878362: Del) that was significantly more frequent among POAG cases than controls (0.78 versus 0.69, p=0.01). The presence of the risk haplotype (arginine/Del) was associated with a 1.6 fold significant increased risk of developing glaucoma (95%CI: 1.14-2.23, p=0.01). We also examined rs1800371 (exon 4: Ser47Pro) as it is a non-synonymous SNP in the same exon as rs1042522, but did not detect a carrier for the rare proline allele in our cohort.

**Table 4:** Genotypic and allele frequencies in POAG patients and controls for rs17878362.

**Genotype/Allele**	**Controls**	**All POAG**	**POAG-HTG**	**POAG-NTG**
Del/Del	0.70	0.76	0.77	0.74
Del/Ins	0.26	0.22	0.21	0.26
Del/Ins	0.04	0.02	0.02	0.00

Del	0.83	0.87	0.87	0.87
Ins	0.17	0.13	0.13	0.13

rs17878362 **/rs1042522**	**Controls**	**All POAG**	**POAG-HTG**	**POAG-NTG**
Del/G	0.69	0.78	0.77	0.81
Del/C	0.14	0.10	0.11	0.07
Ins/G	0.02	0.02	0.01	0.03
Ins/C	0.15	0.10	0.11	0.09

## Discussion

It is well recognized that POAG is a multi-factorial disease and it is expected that multiple genes will contribute to its susceptibility. The goal of this study was to examine the role of the *p53* Arg72Pro SNP (rs1042522) in susceptibility to POAG. In our cohort, we have detected evidence for an association between the arginine variant and increased risk of POAG. This association was not affected by differences in gender or age between cases and controls in our sample. The inclusion of rs17878362 (an Ins/del polymorphism located in intron 3) did not result in a haplotype that had a stronger effect on POAG risk than the Arg72Pro coding SNP located in exon 4. In this study, patients and controls were matched for ethnicity to avoid confounding due to population stratification. All subjects were Caucasian and a majority resided in the same geographical location and represent the Appalachian population. Moreover, the allele frequencies for the Arginine and Proline variants in our controls were similar to the allele frequencies in Caucasian samples from the Hapmap data.

Our results are in contrast to the initial study by Lin et al. [15] where increased frequency of the proline allele (rs1042522) was detected in POAG cases compared to controls. One of the reasons for the discrepancy may be the difference in ethnic populations in each study. According to the Hapmap data, the frequency of the arginine and proline allele are 0.51 and 0.49, respectively, in the Chinese population, and 0.77 and 0.23 in the Caucasian population of European origin. The allele frequencies among controls in our cohort are similar to those from the Hapmap data. Although Acharya et al. [16] did not detect a significant difference in an Indian population; they observed a trend for increased frequency of the arginine allele (rs1042522) and the Del allele (rs17878362). Interestingly, in a Caucasian population from England, Ressiniotis et al. [18] observed increased frequency of the arginine allele in POAG cases compared to controls in subjects with the 16 bp insertion allele (rs17878362). However, the finding of the lack of an association between this SNP (rs1042522) and POAG in the sample by Dimasi et al. [17], which is contrary to our results can not be easily explained.

p53 is one of the key regulators of apoptosis. It can either arrest cell cycle progression in the late G1 phase, thus allowing the DNA to be repaired before replication, or induce apoptosis, leading to cell death. This polymorphism occurs in the proline-rich domain of p53, which is necessary for the protein to fully induce apoptosis. Dumont et al. [19] found that the Arg72 variant had up to 15 fold increased apoptotic ability compared with the Pro72 variant in both inducible cell lines and cells with endogenous p53 homozygous for each variant. They suggested that at least one source of this enhanced apoptotic potential is the greater ability of the Arg72 variant to localize to mitochondria; this localization was accompanied by release of cytochrome c into the cytosol. Another study had noted that Pro72 variant was a stronger inducer of transcription than the Arg72 variant, whereas the Arg72 variant induced apoptosis faster and was a more potent suppressor of transformation than the Pro72 variant [20]. Thus, it is possible that the finding of an association between increased risk of POAG in those homozygous for the Arg72 variant may be due to increased susceptibility of retinal ganglion cells to apoptosis. Interestingly, Dimasi et al. [17] had suggested that a genetic mechanism favoring apoptosis would have a greater role in NTG in which raised IOP is not present. In our cohort, we observed even higher frequencies of the Arginine allele in POAG cases with NTG compared to HTG although this must be interpreted with caution as the number of NTG cases is relatively small.

An additional independent evidence for involvement of *p53* in development of POAG comes from studies of zebrafish mutants lacking functional alleles of *WDR36*, a gene in which mutations are associated with small number of POAG cases. In these mutants, increase expression of a specific *p53* isoform was detected along with the observation that loss of *WDR36* function lead to activation of the p53 stress–response pathway [21].

Further studies consisting of large independent samples with sufficient statistical power for detection of an association and a more detailed analysis of genetic variations within *p53* are warranted in order to determine if the association is with Arg72Pro or other alleles in linkage disequilibrium and to determine its role in the pathogenesis of POAG.
